# Shear Performance of RC Beams Strengthened with High-Performance Fibre-Reinforced Concrete (HPFRC) Under Static and Fatigue Loading

**DOI:** 10.3390/ma17215227

**Published:** 2024-10-27

**Authors:** Xiangsheng Liu, Georgia E. Thermou

**Affiliations:** 1 Centre for Structural Engineering and Informatics, Department of Civil Engineering, The University of Nottingham, Nottingham NG7 2RD, UK; 2 Structural Engineering, Department of Civil Engineering, The University of Nottingham, Nottingham NG7 2RD, UK; georgia.thermou@nottingham.ac.uk

**Keywords:** reinforced concrete, beam, HPFRC, fibre-reinforced concrete, shear strengthening, retrofitting, fatigue

## Abstract

This study experimentally assessed the shear performance of reinforced concrete (RC) beams strengthened with U-shaped High-Performance Fibre-Reinforced Concrete (HPFRC) under static and fatigue loading. Key parameters included HPFRC jacket thickness and beam shear span–depth (*a*/*d*) ratio. Five beams were tested under static loads to determine ultimate shear strengths, followed by fatigue tests on identical beams at 30–70% of ultimate shear strengths at 4 Hz. In static loading experiments, all the HPFRC jacketing proved effective, increasing the shear strength of RC beams by 95% to 130%. Although the strengthening system did not change the failure mode of the beams, the strengthened beams exhibited pseudo-ductile behaviour. As the *a*/*d* increased, the shear enhancement capability of the HPFRC jackets decreased. In fatigue loading experiments, all the HPFRC systems improved the fatigue life of RC beams. Specifically, in beams with an *a*/*d* ratio of 2.0, the fatigue life was extended from 75 cycles to a maximum of 951 cycles, while in beams with an *a*/*d* ratio of 3.5, it increased from 12,525 cycles to 48,786 cycles. In addition, a predictive model has been developed for the fatigue life of HPFRC/UHPFRC shear-strengthened beams, utilising the maximum fatigue load and the design’s ultimate shear strength under static loading conditions.

## 1. Introduction

Due to increased loads, deteriorating environmental conditions, and more stringent design specifications, the existing reinforced concrete (RC) structures have experienced a decline in their load-bearing capacity. They may no longer adequately meet operational requirements. The damage caused by earthquakes over the years has shown the vulnerability of the existing structures to seismic activity [1,2]. For example, the devastating 7.8 magnitude earthquake in Turkey and Syria in January 2023 resulted in over 50,000 deaths and 173,000 collapsed buildings [3]. Most RC buildings constructed before the 1970s have structural deficiencies related to insufficient transverse reinforcement, inadequate lap splice and anchorage lengths, brittle stirrups, and material degradation due to exposure to aggressive environmental conditions [4,5,6,7]. These deficiencies significantly increase the risk of abrupt shear failures and collapses during earthquakes. Therefore, it is crucial to implement structural rehabilitation and strengthening measures to enhance the resilience of older RC buildings against future seismic events [8,9,10].

In addition, the existing structures, especially bridges and coastal structures, are prone to fatigue loading, leading to structural degradation and potential failure over time [11]. The fatigue performance of these structures is crucial for their long-term durability and safety. For example, studies by Laterza et al. [12] and Roberts et al. [13] have explored the fatigue behaviour of masonry and brick arch bridges, highlighting the challenges of maintaining structural integrity under repeated cyclic loading. These studies emphasise the importance of understanding fatigue strength to ensure the safety and service life of the existing infrastructure. Specifically, the durability of RC structures decreases under repeated loading, and deformation and cracks propagate with increasing loading cycles [14,15,16]. This makes embedded steel bars more easily exposed to harsh environments, affecting the structural load-bearing capacity and integrity [14]. In recent years, High-Performance Fibre-Reinforced Concrete (HPFRC) and Ultra-high-Performance Fibre-Reinforced Concrete (UHPFRC) have emerged as promising solutions for structural strengthening [17,18]. The characteristics of ordinary concrete include lower tensile strength and ductility, which can be enhanced by incorporating steel fibres into the matrix [18]. UHPFRC and HPFRC exhibit remarkably similar mechanical properties [19]. They utilise fine powders (silica fume), low water–binder ratios, and superplasticisers, resulting in a dense matrix with enhanced homogeneity and lower permeability than conventional concrete [19,20,21,22,23]. The reduced permeability enhances resistance against the ingress of harmful chemicals, leading to superior corrosion resistance and durability [24,25,26]. HPFRC/UHPFRC exhibits excellent resistance to damage from freeze–thaw cycles and high temperatures, and the shrinkage can be minimised to zero through proper heat treatment, thus enhancing durability against ageing [24,27]. In addition, the low coarse aggregate content and high fibre fraction (typically around 2%) improve the stiffness and ductility of structures [27,28]. HPFRC/UHPFRC demonstrate superior compressive strengths over ordinary concrete, with HPFRC values spanning 90–120 MPa and UHPFRC exceeding 120 MPa [18,24,25,26,29]. Therefore, these properties make HPFRC/UHPFRC an ideal material for strengthening beams as a jacketing device.

Despite the advantages of HPFRC jackets, research on UHPFRC-strengthened beams remains limited and primarily focused on the performance under static loading [16]. In a recent study [28], the tensile zone of non-stirrup rectangular RC beams was replaced with UHPFRC. The study demonstrated the effectiveness of UHPFRC in shear strengthening through four-point bending tests. It was found that all the UHPFRC-strengthened beams failed at 1.6–2.0 times the shear force of the control beams. Moreover, they illustrated that the bond between UHPFRC and normal concrete was very high, so no additional connectors between those two surfaces were needed. Garg et al. [30] used a different technology to apply UHPFRC jacketing. In their tests, all the beams were first loaded to 60% of their ultimate capacity, then cut a U-shaped area with a depth of 25 mm and coated with an epoxy resin, and finally, UHPFRC was cast. The results showed that UHPFRC could increase damaged RC beams’ shear capacity, ductility, and energy absorption. However, they further pointed out that the bond strength between the newly cast UHPFRC and ordinary concrete and the confinement provided by UHPFRC were important factors in enhancing the initial behaviour of stressed RC beams. They recommended improving the interfacial bond through surface treatment techniques to further increase the shear-strengthening performance. He and Liu [31] proposed design guidelines for UHPFRC-strengthened rectangular RC beams that considered size effects. To verify the accuracy of the formula, they tested beams with shear span ratios (*a*/*d*) of 2.2, 2.6, 2.7, and 3.2, respectively. The results proved the influence of the shear span ratio on the shear-strengthening effect and demonstrated the interaction between the UHPFRC layer and internal reinforcement. In summary, research on the shear behaviour of HPFRC/UHPFRC-strengthened beams is limited, especially on the effects of different shear span ratios and HPFFRC/UHPFRC thickness on the shear strengthening performance.

The current understanding of HPFRC’s fatigue behaviour, especially in the context of shear performance, remains limited [16]. Murthy et al. [16] conducted fatigue tests on damaged beams, which were subsequently retrofitted with 10 mm UHPFRC for flexural reinforcement, exploring the effects of various damage levels (70%, 80%, and 90%) on the beams’ fatigue performance. The tests were performed at a stress ratio of 0.1 and a frequency of 2 Hz. The results demonstrated that UHPFRC effectively restored the beams to their pre-damage state, offering increased fatigue life and comparable ductility to undamaged beams. Similarly, Wang et al. [32] conducted testing on eleven T-shaped beams that were flexurally strengthened through the application of UHPFRC jackets. These beams underwent four-point bending fatigue tests, subjected to frequencies of 2–4 Hz, load ranges spanning from 0% to 80% of their ultimate capacity, and enduring up to 2,000,000 cycles. The findings confirmed the effectiveness of UHPFRC in enhancing fatigue resistance and suggested that increasing UHPFRC thickness improved reinforcement effects. It is evident that research on the fatigue performance of HPFRC-strengthened beams is limited, and systematic studies on the fatigue behaviour of shear-strengthened beams are absent. There is a notable gap in knowledge regarding the fatigue shear behaviour of these materials, highlighting the need for further experimental and analytical studies in this area.

Therefore, to address the research gap in the shear enhancement effects of HPFRC-jacketed beams, this study aims to investigate the shear behaviour of RC beams experimentally strengthened with HPFRC jackets under static and fatigue loading. The main test parameters include the shear span ratio of the beams (2.0 and 3.5) and the thickness of the HPFRC jacket (10 mm and 20 mm). This research will provide evidence for HPFRC as an advanced strengthening technique to enhance critical RC structures’ fatigue shear resistance. This study improves the understanding of the static and fatigue shear performance of UHPFRC-strengthened beams, addressing gaps in the current research. To the best of the author’s knowledge, this study represents the first comprehensive analysis of the fatigue performance of UHPFRC shear-strengthened beams. In addition, digital image correlation (DIC) techniques were used to observe the behaviour of the strengthening system under both static and fatigue conditions, offering deeper insights into deformation patterns and failure mechanisms.

## 2. Experimental Programme

### 2.1. Specimen Details

**Figure 1:**
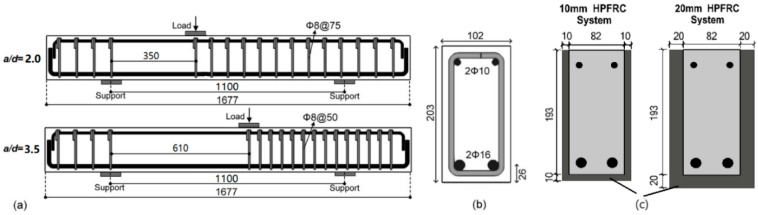
Beam Details: (**a**) Layout of the reinforcement of the beams; (**b**) cross-section; (**c**) strengthening configurations.

The experimental investigation assessed ten RC beams under three-point bending conditions. Out of this set, five beams were subjected to monotonic loading tests, while the remaining five, identical in specifications, were tested under fatigue loading. Within the subset of five beams subjected to monotonic testing, three beams were characterised by a shear span–depth ratio of 2.0 (Group A), and the remaining two had a shear span–depth ratio of 3.5 (Group B). The beams had a rectangular cross-sectional geometry, 102 mm in width, 203 mm in height, and an overall length of 1677 mm, as depicted in Figure 1. The effective span of each beam was 1100 mm. Two shear span lengths were employed: 350 mm for Group A and 620 mm for Group B. No transverse reinforcement was placed within the critical region of the shear span, which was intended to promote shear failure within the beams. In the shear-sufficient span, 8 mm diameter stirrups were placed at 140 mm intervals. Regarding the longitudinal reinforcement, 2 Ø 16 longitudinal rebars were used in tension with 2 Ø 10 rebars in compression. All the beams, except for the two control beams, were strengthened in the shear-deficient regions using U-shaped HPFRC jacketing. This configuration was chosen because the U-shaped jacket effectively confines the sides of the beams, enhancing their shear capacity by targeting diagonal shear cracks that typically develop in critical shear zones [33].

Table 1 summarises the tested beams, which were labelled as ‘X-Y’. ‘X’ denotes the beam group, where ‘A’ indicates the beam with a shear span of 2.0, while ‘B’ indicates the beam with a shear span of 3.5. ‘Y’ corresponds to the beam jacketing configuration, in which ‘C’ represents the control beam, ‘10’ denotes beams with 10 mm HPFRC jacketing, and ‘20’ corresponds to beams with 20 mm HPFRC jacketing.

In the HPFRC system, steel fibres were incorporated into the High-Performance Concrete (HPC) with a volume fraction of 1.66%. According to the manufacturer, the 28-day standard compressive strength and elastic modulus of the HPFRC can reach 106.5 MPa and 43 GPa, respectively. The geometric and mechanical properties of HPC [34] are detailed in Table 2, while Table 3 summarises the properties of the steel fibre [35], with all the data sourced directly from the manufacturers. The average cube compressive strength of the concrete 28 days after casting was 22 MPa. Three 150 mm concrete cubes were tested on the day of beam testing to determine the concrete compressive strength, and the average value was taken. Table 4 and Table 5 present the concrete compression strength for each specimen under monotonic and fatigue loading, respectively. The yield stresses for the longitudinal bars with 16 mm and 10 mm diameters were 538 MPa and 527 MPa, respectively, while the yield stress of the 8 mm-diameter stirrups was 340 MPa.

**Figure 2:**
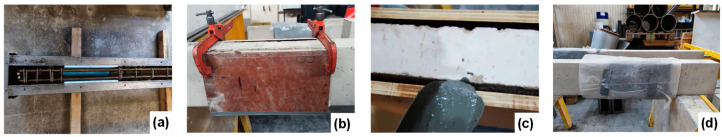
Steps of the hybrid jacketing application: (**a**) create a grooved beam; (**b**) fix the jacketing mould; (**c**) pour HPFRC; (**d**) cure.

Figure 1c illustrates two strengthened configurations: HPFRC jackets with 10 mm and 20 mm thicknesses. All the beams except the four control beams were strengthened in the shear critical zone. Grooves with a depth equal to the cover thickness (10 mm) in the shear critical area of the retrofitted beams were created (Figure 2a). The grooved surface was then roughened, cleaned, and water-saturated before HPFRC was applied. Next, the jacket’s mould was put in place in the critical zone, and the HPFRC was poured (Figure 2b,c). The mould was removed after two days, and the beams were covered with plastic film and cured until the testing time (Figure 2d).

### 2.2. Test Setup

**Figure 3:**
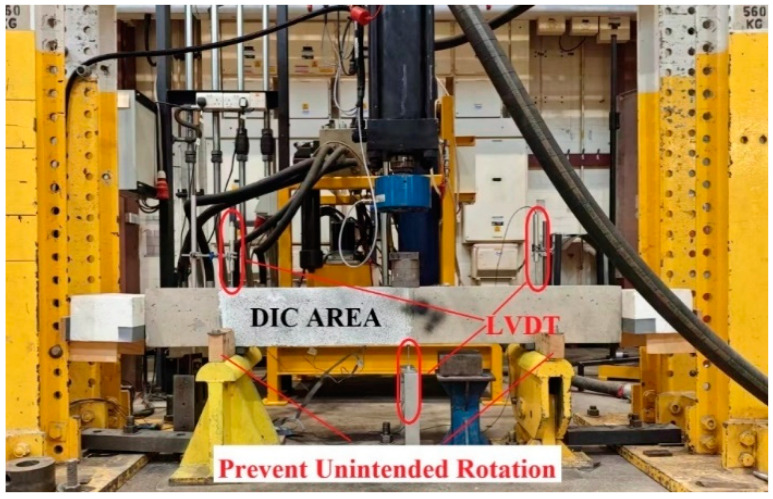
Three-point bending test setup of beams.

Following the test configuration depicted in Figure 3, all the beams were tested under three-point bending by a stiff steel reaction frame featuring a vertically positioned servo-hydraulic actuator with a 500 kN capacity. The beams were positioned on two steel supports anchored to a solid floor using threaded rods. Additional constraints were imposed at both ends of the beams to prevent unintended rotation during fatigue testing. Monitoring the vertical displacement during loading involved using an external Linear Variable Differential Transducer (LVDT) at the load application point. Two LVDTs were positioned at the beam supports to monitor settlement. In addition, digital image correlation (DIC) technology was employed to capture the strain distribution across the shear critical zone in the tested beams.

Static loading tests: The tests were conducted in displacement control mode at 0.02 mm/s. A high-speed camera captured images of the critical shear region every 2 s for subsequent DIC analysis.

Fatigue loading tests: The beams were subjected to cyclic loading until failure, with a maximum cycle limit of 2 million cycles [11]. If the beams did not fail under fatigue loading, subsequent monotonic loading tests were conducted until the failure of the beams. The applied fatigue loads were determined based on the shear resistance ($P_{max}$) of beams with the same strengthening configuration under monotonic loading conditions [11,36,37]. Following the guidelines from fib 2001 [38], the fatigue load range ($P_{l}$ to $P_{h}$) was calculated as $P_{l}=P_{mean}(1-DAF)$ and $P_{h}=P_{mean}(1+DAF)$, where DAF represents the Dynamic Amplification Factor (DAF), ranging from 0.25 to 0.4, and $P_{mean}$ corresponds to 50% of $P_{peak}$. In this project, a DAF of 0.4 was chosen, resulting in a fatigue load range of 30% to 70% for each beam. During fatigue testing, a frequency of 4 Hz was applied to all the specimens to simulate the realistic conditions experienced by traditional RC structures [39,40,41,42]. This frequency was selected to mitigate hysteresis effects, allow for full recovery between cycles, and avoid undesirable heating [43,44]. For digital image correlation (DIC), photos were captured at a frequency of one image every 8 cycles for the initial 10,000 cycles, followed by recordings every 1000 cycles [45]. Subsequently, the high-resolution speckle images were analysed using DIC software (ZEISS INSPECT Correlate 2023, ZEISS, Oberkochen, German).

## 3. Results and Discussion

### 3.1. Static Tests

Table 4 summarises the test results such as the peak load ${(P}_{max})$ and the corresponding displacement ${(\delta }_{max})$; the strength increase in the retrofitted beams $({\Delta P}_{max}=\left(P_{RET}-P_{CON}\right)/P_{CON}$; where $P_{RET}$ and $P_{CON}$ are the peak load of the retrofitted and the corresponding control beam); the ultimate load $P_{u}$ = (80% $P_{max})$ and the corresponding displacement δu; the shear strength of the critical shear span (V; for the control specimen it is equal to $V_{CON}$; for the retrofitted specimens it is equal to $V_{RET}$); the shear strength provided by the strengthening system $V_{JAC}($= $V_{RET}-V_{CON}$; where $V_{RET}$ and $V_{CON}$ are the shear strength of the retrofitted and the corresponding control beam); the displacement ductility $(\mu_{\delta }$); and the failure mode. The ‘SH’ in failure mode corresponds to shear failure.

**Figure 4:**
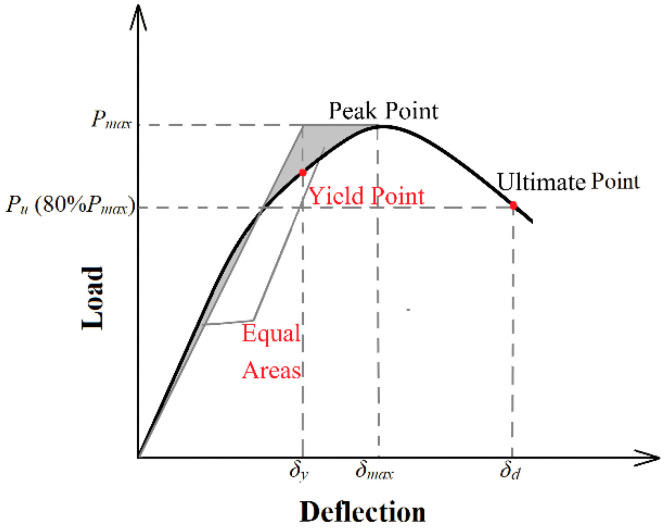
Determination of yield and ultimate point.

The displacement ductility ($\mu_{\delta }$) of a structural element quantifies its deformation capacity and is defined as the ratio of displacement at ultimate load (*δ_u_*) to the displacement corresponding to the yield load (*δ_y_*) [46]. In accordance with the recommendations outlined in ASCE/SEI Standard 41-06 [47], the displacement ductility was conceptually formalised by approximating the experimental load–deflection curve with a bilinear model. As depicted in Figure 4, the yield point for non-ideal elasto-plastic elements was identified based on the energy equivalence method, which subsequently facilitated the derivation of the displacement ductility for the tested samples [46]. The ductility indices were not calculated for control beams A-N and B-N because they did not exhibit ductile behaviour.

**Figure 5:**
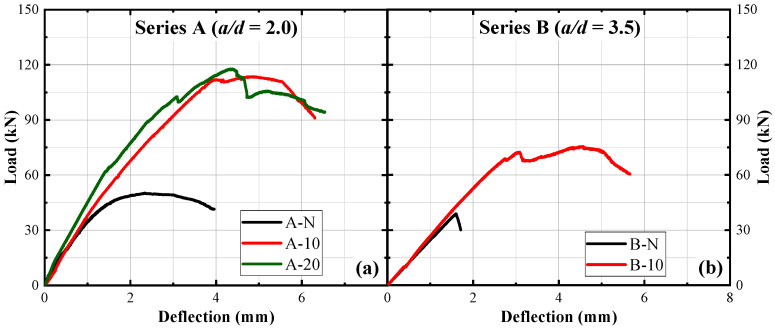
Load–deflection curves under monotonic loading: (**a**) Series A; (**b**) Series B.

The load–deflection curves for the five tested beams under monotonic loading are shown in Figure 5. Regarding Series A (*a*/*d* = 2.0), the control beam failed in shear at a peak load of 51.3 kN (corresponding displacement of 2.5 mm). The HPFRC jacketing has substantially increased the peak load capacity of RC beams. Specifically, beams A-10 and A-20 demonstrated peak load increases of 122% and 130%, respectively, compared to the control beam A-N. This marked improvement is attributed to the fibre ‘bridging effect’, which facilitates the distribution of stress across developing cracks, thereby fortifying the structural integrity and delaying the onset of failure. The 20 mm HPFRC jacketing (A-20) exhibited a higher peak load compared to the 10 mm jacketing (A-10). This enhancement is likely due to the thicker jacket’s ability to mitigate fibre aggregation, leading to improved fibre distribution within the HPFRC matrix and bolstering its reinforcing efficacy. Additionally, the thicker reinforcement layer amplifies the HPFRC system’s influence on the beam’s load–deflection behaviour, increasing stiffness for A-20. In Series B (*a*/*d* = 3.5), the 10 mm HPFRC jacketing (B-10) led to a 48% increase in peak load.

**Table 4:** Summary of monotonic loading test results.

Series	Beam	*f_c_* (MPa)	*P_max_* (kN)	Δ*P_max_* (%)	*P_u_* (kN)	*δ_max_* (mm)	*δ_d_* (mm)	*V* (kN)	*V_JAC_* (kN)	*V_JAC_*/*V* (%)	$\mu_{\delta }$	Failure Mode
A	A-N	26.4	51.3	-	41.1	2.50	3.96	35.0 *	-	-	-	SH
	A-10	30.6	113.7	122	91.0	4.93	6.30	77.5 ^$^	42.5	55	1.72	SH
	A-20	28.4	117.8	130	94.2	4.34	6.53	80.3 ^$^	45.3	56	2.04	SH
B	B-N	24.2	38.8	-	31.2	1.41	1.50	16.9 *	-	-	-	SH
	B-10	26.7	75.5	95	60.4	4.34	5.43	32.9 ^$^	16.0	49	2.06	SH

* $V_{CON}$; ^$^ $V_{RET}$.

**Figure 6:**
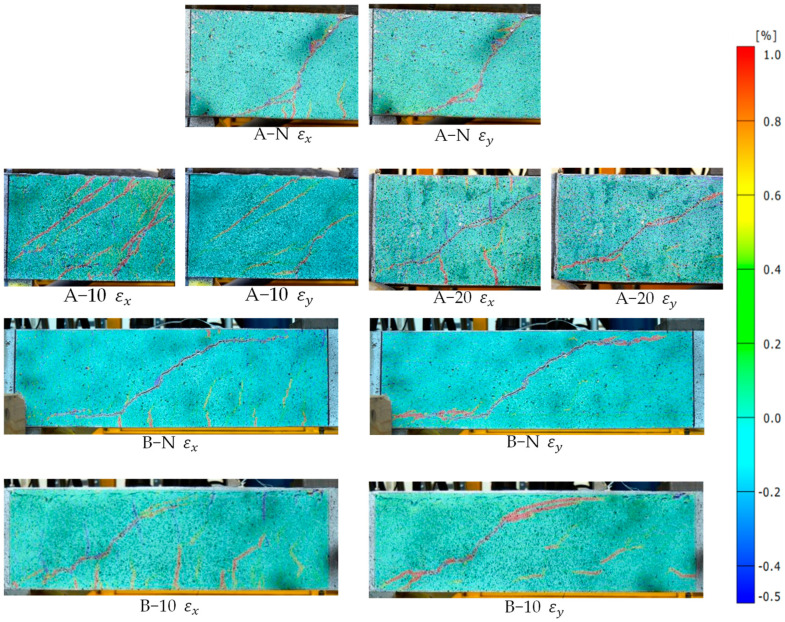
Strain contours in the critical shear span under monotonic loading.

With the escalation of the *a*/*d* ratio (from 2 to 3.5), there was a noticeable decline in *V*, Δ*P_max_* and *V_JAC_*/*V*, highlighting the diminished reinforcement efficacy of HPFRC as *a*/*d* increased. This phenomenon occurs because the beam changes from a deep to a slender shape, shifting the main way it supports weight from an arch-like structure to more of a truss system, which decreases its ability to resist shear forces [48,49]. In detail, for the Series A beams (*a*/*d* = 2, deep beams), arching action dominates after the formation of diagonal cracks. The majority of the load is directly transferred from the point of application to the support via the diagonal strut, indicating that the diagonal strut primarily bears the load [49]. The influence of arching action diminishes with an increase in the *a*/*d* ratio, thereby reducing the contribution of concrete to shear strength. In addition, the larger shear span increases the likelihood of shear damage, impairing the bond between the jacket and substrate, and facilitating detachment [49]. An elevated *a*/*d* ratio also intensifies shear stress concentration near the beam ends, precipitating the early failure of the reinforcement system and reducing shear enhancement, as evidenced by the DIC strain fields in Figure 6.

Figure 6 presents the horizontal and vertical strain fields obtained via DIC for the tested beams at peak load. These results provide detailed insight into strain distribution across the shear span of the HPFRC-strengthened beams. The DIC images reveal a more uniform strain distribution, particularly in critical regions, underscoring HPFRC’s effective stress redistribution capabilities.

As shown in Figure 5, all the strengthened beams demonstrated a pseudo-ductile behaviour and maintained the peak load while the beam deformed due to the ‘bridging effect’. This observation suggests the HPFRC system’s pivotal role in augmenting the energy absorption and dissipation capabilities of the beams, directly impacting the beams’ seismic performance resilience. This ‘pseudo-ductile behaviour’ is characterised by a gradual reduction in load-carrying capacity after the peak load, instead of a sudden brittle failure, due to the strain-hardening properties of HPFRC. The fibres within the HPFRC matrix bridge crack, allowing the material to sustain loads even after initial cracking, which enhances the beam’s energy absorption and provides a more controlled failure mode [50]. Such behaviour is particularly beneficial for seismic applications, as it allows structures to absorb and dissipate seismic energy more effectively. From Table 4, the displacement ductility range of the strengthened beams lies between 1.72 and 2.06. Furthermore, the ductility increases with the augmentation of the UHPFRC jacket thickness and the ratio of *a*/*d*. This observation not only underscores the enhanced potential of the reinforcement system attributable to the increased thickness, but also validates the influence of altered load transfer mechanisms, resulting from variations in *a*/*d*, on ductility.

**Figure 7:**
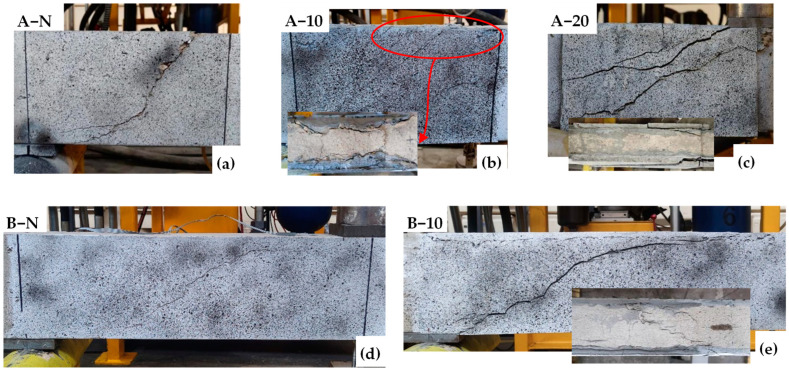
Crack patterns and failure modes under monotonic loading: (**a**) A-N; (**b**) A-10; (**c**) A-20; (**d**) B-N; (**e**) B-10.

The condition of all the tested beams at the end of testing is depicted in Figure 7, where the damage is localised in the shear critical region. The control beams (A-N and B-N) exhibited a typical diagonal tension failure in the shear span joining the points of load application and support (see Figure 7a,d). In Series A, the HPFRC-reinforced beams, A-10 and A-20, demonstrated similar failure modes (Figure 7c,d), namely shear detachment failure with several diagonal shear cracks forming in the critical region. The observed gradual failure further corroborates that the strengthened beams exhibit pseudo-ductility. Additionally, the enhancement in ductility optimises the stress–strain transfer mechanism within the beams, resulting in multiple cracks on the HPFRC surface, as opposed to a single crack. The 20 mm HPFRC system displayed enhanced crack visibility and jacket detachment. Despite improved adhesion due to superior fibre distribution within the 20 mm HPFRC jacket, the marked stiffness contrast with the substrate resulted in a divergent load–stress response, exacerbating detachment issues relative to A-10. In the context of a higher *a*/*d* (3.5), B-10 mirrored the control beam’s failure mode. This is because the beam changes to slender beams, leading to uneven stress distribution and a predisposition for cracks to propagate along singular paths of weakness [51]. Consequently, the capacity of HPFRC to induce multiple cracking is mitigated, diminishing its potential to alter failure dynamics significantly.

### 3.2. Fatigue Tests

The fatigue test results are summarised in Table 5, including the concrete compressive strength of all the beams on the test day ($f_{c}$), the fatigue life ($N_{f})$, the total deflection (δ), the stiffness degradation (β) at the last cycle, and the energy dissipation ($\Psi_{d}$) at the last cycle, as well as the failure mode. The stiffness degradation for each cycle can be calculated as follows:

(1) $$\beta =\frac{K_{1}-K_{n}}{K_{1}}\times 100\%$$

where $K_{n}$ is the stiffness of each cycle, and $K_{1}$ is the stiffness of the first cycle. The stiffness (*K*) for each cycle is calculated as the ratio of the load range ($P_{max}-P_{min}$) to the corresponding displacement range ($\delta_{max}-\delta_{min}$) [40]. The energy dissipation ($\Psi_{d}$) is represented by the area enclosed in the load–displacement curve for each cycle: the difference between the energy absorbed during the load increase phase ($\Psi_{a}$) and the energy released during the load decrease phase ($\Psi_{r}$) [52]. Furthermore, as previously stated, the fatigue load range encompasses a span of 30% to 70% of $P_{peak}$. Consequently, based on the outcomes of static testing, the loads applied to each beam are also delineated in Table 5.

**Table 5:** Summary of fatigue loading test results.

Series	Beam	$P_{l}$ (kN)	$P_{h}$ (kN)	$f_{c}$ (MPa)	$N_{f}$ (cycles)	δ (mm)	β (%)	$\Psi_{d}$ (kN·mm)	Failure Mode
A	A-N	15.4	35.9	24.7	75	3.81	56.0	8.5	SH
	A-10	34.1	79.6	23.7	90	4.34	25.2	11.8	SH
	A-20	35.3	82.5	31.2	951	6.99	47.8	13.6	SH
B	B-N	11.6	27.2	24.4	12,525	1.61	30.4	2.56	SH
	B-10	22.7	52.9	26.9	48,786	4.79	48.8	7.42	SH

The results indicate that HPFRC jacketing enhanced the fatigue life and maximum deflection of shear-deficient beams, irrespective of their classification as Series A or B. Sample A-10 showed a fatigue life similar to that of the control beam, but it also demonstrated a 20% increase in the number of cycles. However, given the significantly higher load applied to A-10 in comparison to the control beam, it can be contended that the HPFRC system in A-10 maintained its effectiveness. Within Series A, increasing the thickness of HPFRC from 10 mm to 20 mm resulted in a substantial improvement in the fatigue behaviour of the RC beams, with the fatigue life increasing by almost 10 times. This improvement is attributed to the superior fibre distribution achieved with the thicker jacket, which aligns with the observations from static loading tests. Furthermore, as the *a*/*d* ratio increased from 2.0 to 3.5, the performance of the 10 mm strengthening system also improved. In Series B, B-10 exhibited even more significant improvements in fatigue life (four times) and maximum deflection than A-10, which is not only due to the different load-carrying mechanisms arising from varying *a*/*d* but also because the extended coverage of HPFRC provides a more pronounced enhancement in fatigue performance.

**Figure 8:**
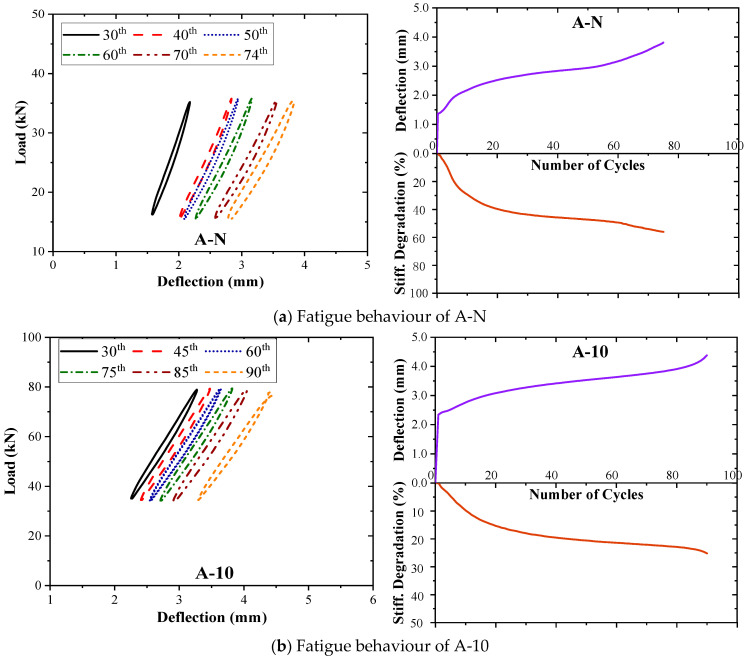

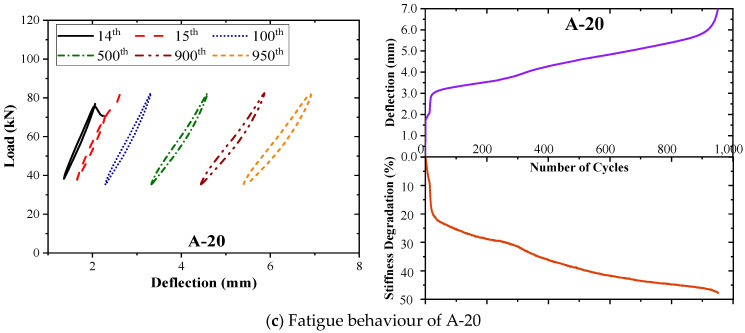
Fatigue behaviour of Series A tested beams: (**a**) A-N; (**b**) A-10; (**c**) A-20.

**Figure 9:**
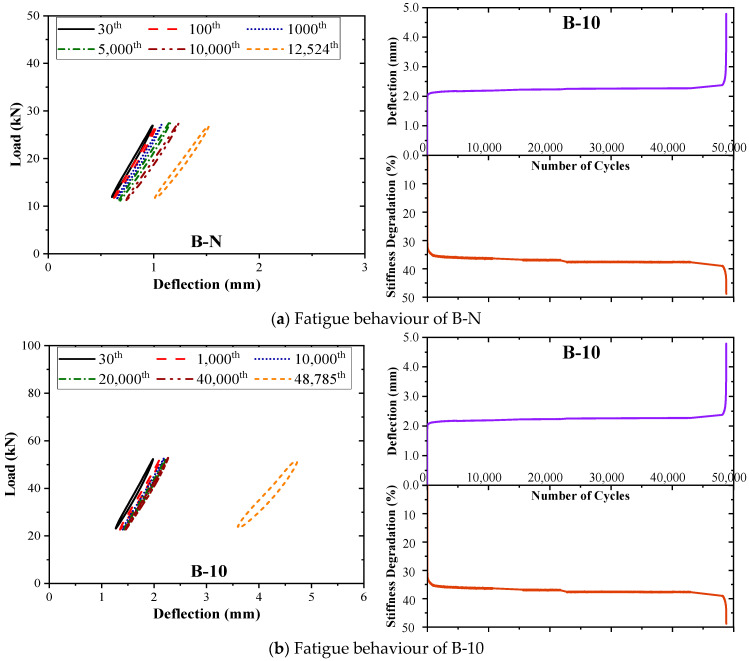
Fatigue behaviour of Series A tested beams: (**a**) B-N; (**b**) B-10.

Figure 8 and Figure 9 present the load–displacement response, as well as the displacement (*δ*) at cap P sub h and stiffness degradation (*β*) with an increase in the number of cycles under the fatigue of the control beam and the strengthened beams. The left plots show the load–deflection behaviour for various cycle intervals up to the cycle just before failure. As the applied loads stabilised by the 30th cycle, the load–displacement curves for all the specimens are shown from this cycle. Beam A-20 exhibited significant cracking during the 14th cycle but continued normal operation after the 15th; thus, its curve starts from the 14th cycle to capture this change. The various outcomes of *δ* and *β* with the number of cycles are demonstrated on the right plots. In general, the comprehensive response of all the tested beams demonstrates a typical pattern of damage accumulation under fatigue loading, echoing the behaviour exhibited by typical reinforced concrete (RC) beams in numerous prior studies [11,39,53,54]. The specimens exhibited an accelerated rate of damage propagation, characterised by three stages throughout the overall response:

- Initial stage: During the initial cycles, the deflection and strain of various components, including the longitudinal reinforcement, strengthening system, and concrete, underwent a rapid increase due to the emergence of a significant number of initial cracks.
- Stable stage: The response shifted into a stable phase marked by a notable deceleration in the accumulation of damage. During this stage, the rate of increase in deflection and the degradation of stiffness decelerated. This stable phase persisted until the commencement of the final, brief stage preceding failure.
- Final stage: The tested beams experienced a sudden and marked surge in both deflection and stiffness degradation, ultimately leading to failure.

These stages encapsulate the characteristic behaviours exhibited by the beams as they undergo progressive damage accumulation under fatigue loading. In Series A, a notable decrease in the slope of the load–deflection curves was observed for the control beam A-N, with *β* reaching 56.0% at failure. However, the application of HPFRC significantly slowed down the rate of stiffness degradation in the beams, with *β* values of 25.2% and 47.8% for A-10 and A-20 at failure, respectively. This indicates that the rate of damage accumulation in the strengthened beams decreased, enabling better resistance to structural deformation. A-20 beam exhibited a more pronounced increase in deflection and stiffness degradation during the ‘stable stage’ compared to other beams due to premature crack formation that expedited the damage progression and thus partially negated the fatigue performance enhancement provided by HPFRC. This phenomenon may be attributed to imperfections in the reinforcement process. In Series B, the amplitudes of deflection and stiffness degradation during the final stage were higher for B-N and B-10 compared to Series A. This suggests that an increase in the *a*/*d* led to a slower rate of damage development during the stable stage, resulting in smoother curves and extended fatigue life.

**Figure 10:**
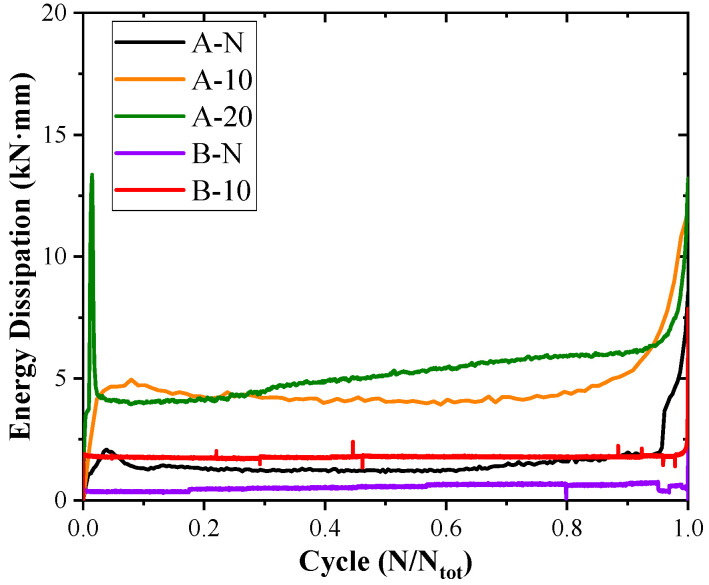
Energy dissipation verse cycles curves.

The energy dissipation ($\Psi_{d}$) versus normalised cycles for each beam is presented in Figure 10. The energy dissipation ($\Psi_{d}$) was calculated for each cycle as the area enclosed by the load–deflection hysteresis loop during cyclic loading. To facilitate comparison, the cycle numbers for all the beams were normalised, i.e., expressed as a ratio of the cycle number (*N*) to the total number of cycles (*N_tot_*). A three-stage trend similar to that observed in *δ* and *β* was noticed in the $\Psi_{d}$ measurements of the tested beams. A consistent three-stage trend was observed in the $\Psi_{d}$ measurements, except for beam A-20, which showed significant energy dissipation at the boundary between the ‘initial’ and ‘stable stages’, correlating with crack emergence in its 14th cycle. Due to the higher loads experienced by the reinforced beams, all of them exhibited greater $\Psi_{d}$ values compared to the control beam. Furthermore, an increase in the shear span–depth ratio resulted in reduced energy loss per cycle for the beams, potentially enhancing their fatigue resistance.

**Figure 11:**
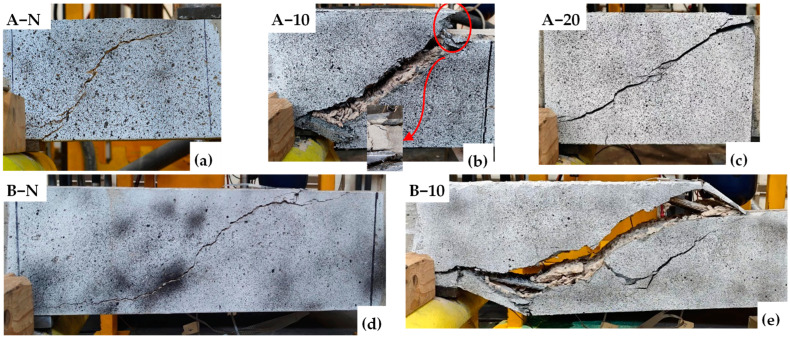
Crack patterns and failure modes under fatigue loading: (**a**) A-N; (**b**) A-10; (**c**) A-20; (**d**) B-N; (**e**) B-10.

**Figure 12:**
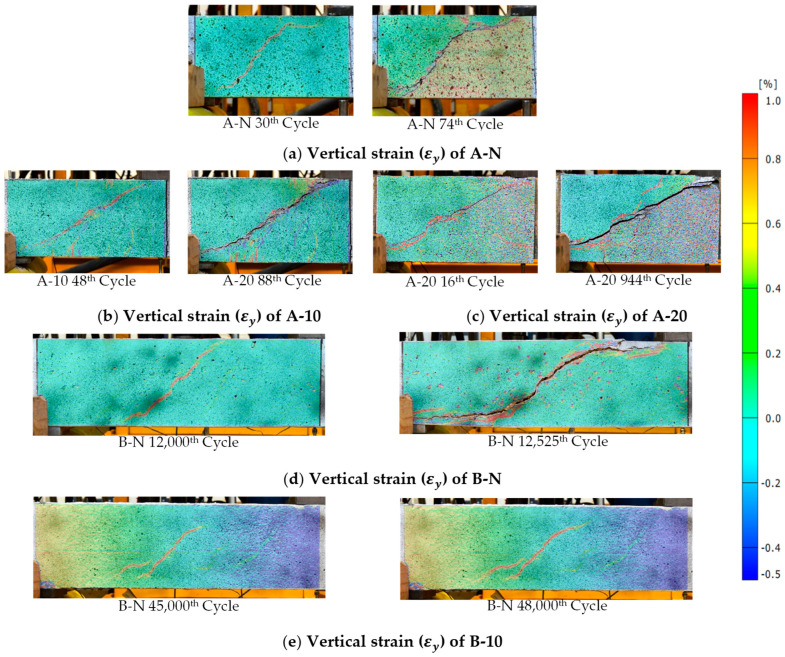
Strain contours in the critical shear span at failure of the beams: (**a**) A-N; (**b**) A-10; (**c**) A-20; (**d**) B-N; (**e**) B-10.

As shown in Figure 11a–e, all the tested beams experienced shear failure. Both the control and strengthened beams in Series A and B exhibited an identical failure mode, characterised by a typical diagonal tensile failure within the shear span between the load application point and the support points. As the number of cycles increased, the gradual accumulation of damage led to a progressive decline in the shear capacity of the specimens. This degradation culminated in the formation of a through-diagonal crack within the shear insufficient zone, ultimately resulting in beam failure. Prior to failure, a significant increase in beam deflection was observed. Additionally, due to the stiffness difference between the strengthening system and the substrate, their stress–strain responses varied, leading to the partial jacketing detachment at failure (as shown in Figure 11b). To gain a deeper understanding of the damage progression in the tested beams, vertical strain ($\varepsilon_{y}$) distributions were analysed using DIC at different cycles, as depicted in Figure 12. Among them, although lacking visible cracks at the initial cycle, exhibited strain patterns in the mid-section of the shear-deficient region that closely resembled the final inclined shear crack. The presence of HPFRC optimised strain distribution, making it more dispersed compared to control beams. Increasing HPFRC thickness and the shear span–depth ratio also enhanced ductility, as indicated by the overall increase in vertical strain.

## 4. Fatigue Life Prediction of the HPFRC Jacketed Beams

*S-N* curves are widely used to assess the fatigue life of concrete structures [53,55,56]:

(2) S=A−αLogN

where *S* refers to the cyclic stress level. A and α are the parameters calculated by the least squares method based on the experimental data. Since no stirrups were arranged in the reinforcement zone of the tested beams, the stress amplitude of the stirrups cannot be calculated. Therefore, ‘*S*’ can be represented by the fatigue shear strength attenuation coefficient of the inclined section [53,55,56]:

(3) $$S=\frac{\sigma_{max}}{f_{fu}}=\frac{P_{h}}{P_{ref}}$$

where $\sigma_{max}$ is the maximum applied fatigue stress; $f_{fu}$ is the ultimate strength of the RC beam; $P_{h}$ is the applied maximum fatigue load; and $P_{ref}$ is the design static shear ultimate strength of beams.

**Figure 13:**
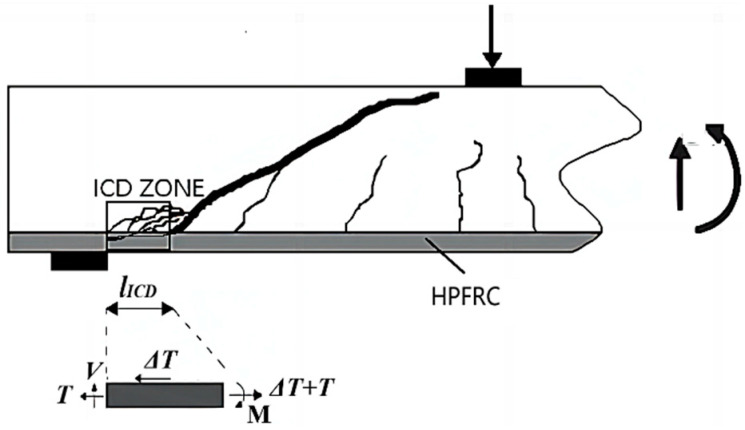
Fatigue shear failure diagram for bottom bonded HPFRC-RC beam.

Currently, research on predicting the shear strength of HPFRC/UHPFRC jacketed beams is limited, with the existing models primarily focused on bottom-bonded strengthening configurations [31,57]. As shown in Figure 13, the bottom bonding system exhibits delayed cracking upon failure, resulting in the formation of an intermediate crack-induced debonding (ICD) zone between the crack initiation point and the support. Within this zone, high shear stress leads to the development of numerous diagonal flexural–shear cracks, while the pry-out stress also reaches its maximum, causing the premature detachment of the strengthening system’s ICD zone. Based on these, Refs. [31,57] proposed a prediction model that considers the length of the ICD zone and the bending moment of the UHPFRC layer. However, in the U-shaped configuration, the side parts integrated with the bottom part prevent the formation of such a zone, rendering this model inapplicable. Furthermore, the relevant design codes primarily focus on the specification of HPFRC/UHPFRC as structural elements, with a lack of guidance on their use as reinforcement systems. The authors of [58,59] have applied the shear design model for HPFRC proposed by the Japan Society of Civil Engineers (JSCE) [60] to bottom bonding jacketing. Therefore, this paper adapts this model for predicting the shear strength of the U-shaped HPFRC reinforcement system. Since no stirrups are present in the strengthened area, $P_{ref}$ comprises shear strength contributions from concrete ($V_{c}$) and FRCM jacket ($V_{JAC}$):

(4) $$P_{ref}=V_{c}+V_{JAC}$$

where $V_{c}$ is the shear strength of the substrate, $V_{jac}$ is the shear strength of the strengthening system. The shear strength contributed by the concrete beam is typically calculated using the model in EC2 [61]:

(5) $$V_{C}^{EC2}=0.18k{\left(100\rho_{long}f_{c}^{\prime }\right)}^{1/3}b_{w}d$$

where $f_{c}^{\prime }$ is the compressive strength of concrete obtained from cylinders; *d* is the depth of the cross-section; $\rho_{long}$ is the area ratio of the tensile reinforcement; and $k=1+\sqrt{\left(200/d\right)}\;\leq 2.0$ (with *d* in mm) is a factor that considers the size effect.

In U-shaped jackets, $V_{JAC}$ is combined with three parts: two side parts and one bottom part. The shear strength of each component is determined by isolating the shear contribution from the cementitious matrix and fibre, as per JSCE [60]:

(6) $$V_{FRC}=V_{m}+V_{f}=0.18\sqrt{f_{cf}}b_{wj}d_{j}+f_{vdf}b_{wj}z$$

where $V_{m}$ is shear strength contributions from concrete in HPFRC; $V_{f}$ is shear strength contributions from fibre in HPFRC; $b_{wf}$ and $h_{f}$ are the width and height of each part, respectively; $f_{cf}$ is the compressive strength of HPFRC; $f_{vdf}=0.3f_{cf}^{2/3}$ is the design average tensile strength perpendicular to diagonal cracks; $z=d_{FRC}/1.15$ is the distance from the location of the compressive stress resultant to the centroid of tensile reinforcement for each part; and $d_{FRC}=0.9h_{FRC}$ is the effective depth of each part.

**Figure 14:**
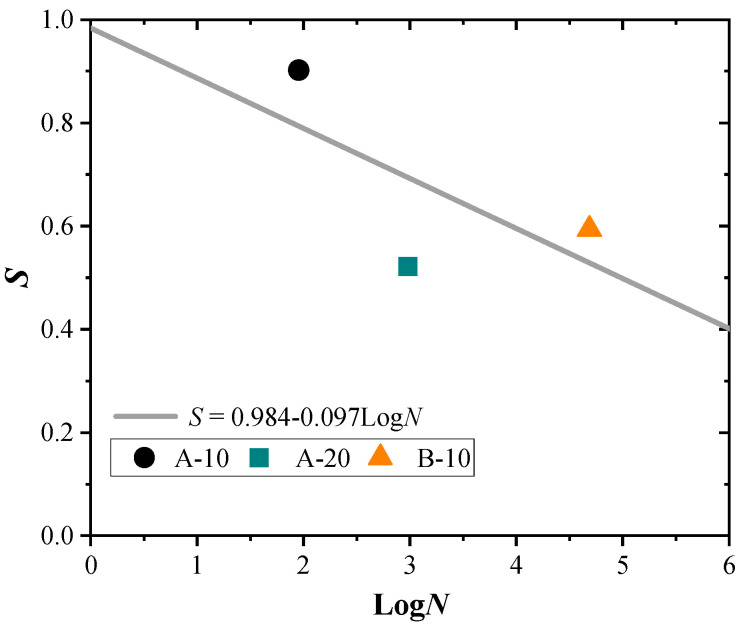
*S-N* relationship of HPFRC strengthened beams.

According to the calculation results, the *S-N* relationship of HPFRC-strengthened beams is depicted in Figure 14, alongside the *S-N* expression derived from the three tested beams. It is worth noting that analysing the *S-N* relationship requires a substantial amount of data [62]. However, as this study is the first investigation into the shear fatigue behaviours of HPFRC-strengthened beams, further analysis is limited by the lack of additional data. Moreover, based on the *S-N* expression, it is expected that an increase in *S* would lead to a gradual decrease in *N*. While this trend holds true for Series A alone, as observed in Figure 13, it appears to be mitigated when considering specimen B-10. This deviation can be attributed to the varying fatigue responses associated with different shear span ratios. Therefore, in the future, with a sufficient amount of data, distinct *S-N* expressions could be derived by grouping the data based on shear span ratios.

## 5. Conclusions

The experimental study presented in this paper investigated the static and fatigue performance of RC beams strengthened with U-shaped HPFRC jacketing, focusing on the effects of HPFRC thickness (10 mm and 20 mm) and shear span–depth ratio (*a*/*d* = 2.0 and 3.5). The key findings are summarised as follows:

- Static Loading: HPFRC jacketing increased the shear strength of RC beams by 95% to 130%. Strengthening effects were more pronounced with increased HPFRC thickness, especially in deeper beams (*a*/*d* = 2.0), while a reduced enhancement was observed as the a/d ratio increased. The strengthened beams exhibited partial detachment but maintained a shear failure mode, with fibre bridging contributing to crack control.
- Fatigue Loading: HPFRC significantly improved the fatigue life of RC beams, extending the number of cycles by up to 951 cycles in beams with *a*/*d* = 2.0, and up to 48,786 cycles in beams with *a*/*d* = 3.5. The typical diagonal tension failure mode persisted across all the specimens. The beams experienced three distinct phases of deflection, stiffness degradation, and energy dissipation, related to damage accumulation.
- Fatigue Life Prediction Model: A predictive model for fatigue life was developed based on maximum fatigue load and ultimate shear strength under static loading. Further validation with additional data are required to refine the model’s accuracy.

These findings demonstrate the potential of HPFRC jacketing as an effective solution for enhancing the shear and fatigue performance of RC beams. It is particularly suitable for retrofitting older RC structures lacking sufficient shear reinforcement, such as bridges, coastal structures, and buildings constructed under outdated design codes. The enhanced shear capacity provided by HPFRC jacketing makes it an effective method for improving the resilience of these structures against static and cyclic loads, including seismic events and fatigue caused by repeated loading. Therefore, this technique contributes to extending the service life of these structures and ensuring their safety.

## Figures and Tables

**Table 1:** Details of the specimens.

	Name	Jacketing Type
Group A (*a*/*d* = 2.0)	A-N	Control beam
	A-10	10 mm HPFRC
	A-20	20 mm HPFRC
Group B (*a*/*d* = 3.5)	B-N	Control beam
	B-10	10 mm HPFRC

**Table 2:** Properties of the HPC used according to the manufacturer [34].

	Mixture Density (kg/cm^3^)	$f_{cm}$ ^1^ (MPa)	$f_{f}$ ^2^ (MPa)	$f_{b}$ ^3^ (MPa)	$E_{f}$ ^4^ (GPa)
HPC	2270	110	14	2	34

^1^ $f_{cm}$ = compressive strength (28 d); ^2^ $f_{f}$ = flexural strength (28 d); ^3^ $f_{b}$ = bond strength (28 d); ^4^ $E_{f}$ = elastic modulus.

**Table 3:** Properties of the HPC used according to the manufacturer [35].

	Shape	Diameter (mm)	Length (mm)	$f_{fu}$ ^1^ (MPa)	$E_{f}$ (GPa)	$\varepsilon_{fu}$ ^2^ (%)
Steel Fibre	straight rigid	0.2	13	3100	200	1

^1^ $f_{fu}$ = tensile strength; ^2^ $\varepsilon_{fu}$ = fibre’s strain to failure.

## Data Availability

Dataset available upon request from the authors.
